# Studying the effects of McRoberts and neonate-focused maneuvers on the neonatal brachial plexus during shoulder dystocia

**DOI:** 10.3389/fbioe.2025.1474154

**Published:** 2025-05-01

**Authors:** Joy A. Iaconianni, Rania Bakhri, Bernard Gonik, Sriram Balasubramanian, Anita Singh

**Affiliations:** ^1^ School of Biomedical Engineering, Science and Health Systems, Drexel University, Philadelphia, PA, United States; ^2^ Bioengineering Department, Temple University, Philadelphia, PA, United States; ^3^ Obstetrics and Gynecology—School of Medicine, Wayne State University, Detroit, MI, United States

**Keywords:** shoulder dystocia, neonatal, brachial plexus, maneuvers, birthing, injury

## Abstract

This study investigates the effects of clinical delivery maneuvers on neonatal brachial plexus (BP) during complicated birthing scenarios such as shoulder dystocia. Shoulder dystocia occurs when the anterior shoulder of the neonate is obstructed behind the maternal symphysis pubis and prevents the delivery of the neonate. Maneuvers such as McRoberts, application of suprapubic pressure (SPP), oblique positioning, and posterior arm delivery are performed sequentially to alleviate the obstruction. This study used MADYMO, a computer software program, to simulate these maneuvers during shoulder dystocia while maternal endogenous forces (82N and 129N) were applied. The recorded outcomes were the magnitude of neonatal BP stretch during delivery and the amount of clinician-applied traction (CAT) force, if required, to achieve delivery. The lithotomy position was treated as the baseline and compared to the McRoberts position, at 82N and 129N maternal forces. Additionally, in McRoberts position, at 82N and 129N maternal forces, neonate-focused maneuvers were applied, and the clinician applied traction (CAT) force, if required, to achieve delivery was recorded along with the resulting neonatal BP stretch. The simulations, at 82N maternal force, reported a decrease in required CAT force in the McRoberts position compared to the lithotomy position. The results of the neonate-focused maneuvers reported a further decrease in the CAT force and the resulting BP stretch. Furthermore, increasing SPP from 40N to 100N reported no required CAT force for delivery along with decreased BP stretch. Oblique positioning further decreased the BP stretch, and the posterior arm delivery of the neonate resulted in the least amount of BP stretch. No CAT forces were required during these maneuvers. The simulations, at 129N maternal force, reported similar trends of reduced BP stretch during delivery except no CAT forces were required during any simulated conditions. Findings from this study help understand the effects of McRoberts position and neonate-focused maneuvers on neonatal brachial plexus during complicated shoulder dystocia delivery. The reported required delivery forces, both maternal and CAT also lay the groundwork for clinician training and education while guiding the development of preventative approaches that can limit neonatal injuries.

## 1 Introduction

Shoulder dystocia is an obstetric emergency in which the anterior shoulder of the neonate is obstructed by the symphysis pubis of the maternal pelvis (Ouzounian, 2016; Dash et al., 2018; Menticoglou, 2018). With the persistence of this obstruction, delivery attempts may lead to the overstretching of the neonate’s brachial plexus (BP), thereby resulting in neonatal brachial plexus palsy (NBPP) (Dunbar et al., 2021; Heinonen et al., 2021). BP is a network of nerves that span from the C5 to T1 vertebrae of the spinal cord and throughout both arms and hands (Leinberry and Wehbé, 2004). NBPP occurs in 1–4 in 1,000 vaginal deliveries and leads to temporary or permanent paralysis of the affected arm, known as Erb’s or Klumpke’s palsy (Orozco et al., 2021).

The complication of shoulder dystocia leads to a sequence of maneuvers that are performed by the clinician until delivery of the neonate is achieved (Lok, Cheng, and Leung, 2016). Firstly, the mother is assisted into the McRoberts position from the starting lithotomy position by hyper-flexing her knees to her chest. This effectively rotates the pelvis by approximately 20° and flattens the sacral promontory, allowing for more space for the shoulder width of the neonate within the maternal pelvis (Gonik et al., 2000; Allen, 2007; Lok et al., 2016; Zimerman et al., 2018). If the obstruction persists, the clinician then applies force to the soft tissue of the mother directly superior to the symphysis pubis, known as suprapubic pressure (SPP) (Nocon et al., 1993; Leung et al., 2011). The SPP force application aims to target the obstructed shoulder of the neonate by either compressing the shoulder width or by rotating the neonate into an oblique position (Gurewitsch and Allen, 2005). The oblique position refers to the alignment of the shoulders of the neonate with the largest diameter of the maternal pelvis. This can be achieved by the clinician by applying SPP at an angle as previously mentioned or by locating the anterior shoulder through the vaginal opening and rotating clockwise or counterclockwise, known as the Woods’ screw or Rubin’s maneuvers (Mazzanti, 1959; Gurewitsch and Allen, 2005). If delivery remains unsuccessful, posterior arm delivery, which is a more invasive maneuver, is performed. During the Posterior arm delivery, the neonate’s posterior arm is located through the vaginal opening by the clinician and delivered before the shoulders of the neonate thus reducing the shoulder width of the neonate (Poggi, 2003; Allen, 2007). Although posterior arm delivery is associated with higher rates of humeral or clavicle fracture, previous modeling studies have shown it to also be associated with the greatest reduction in BP stretch (Gonik et al., 2003a; Gonik et al., 2003b; Grimm et al., 2010; Zimerman et al., 2018).

The focus of this study was to investigate the effects of the discussed maneuvers on the force required to achieve delivery and the resulting neonatal BP stretch. Delivery forces, both endogenous and exogenous, and the resulting neonatal BP stretch are difficult to measure clinically due to technical complications and ethical limitations. Computational modeling serves as a promising alternative tool that can be utilized to address the technical and ethical limitations of studying neonatal BP stretch clinically. MADYMO is the computational modeling software used in this study with the maternal pelvis and neonate models adapted from previous work (Gonik et al., 2003a; Gonik et al., 2003b; Grimm et al., 2010; Zimerman et al., 2018). The developed MADYMO models were used to simulate maternal and infant maneuvers while replicating clinical shoulder dystocia delivery scenarios. The simulations reported the effects of these applied maneuvers on the neonatal brachial plexus while reporting the need and the amount of clinician-applied force needed to achieve delivery.

## 2 Materials and methods

MAthematical DYnamic MOdeling (MADYMO) (v2020.2, Siemens Inc., OR), a modeling software that utilizes multi-body systems, was used to create and simulate the Lithotomy, McRoberts and neonate-focused maneuvers associated with shoulder dystocia. MADYMO has the capability to apply various parameters such as position, force, acceleration, and contact characteristics to any 3-dimensional rigid bodies and can output data such as contact force, displacement, velocity, and elongation. For this study, maternal pelvis and neonate models were used to investigate the effects of shoulder dystocia and various maneuvers on neonatal brachial plexus while maternal forces (82N and 129N) were applied during delivery. The clinician-applied traction delivery force was also investigated to achieve delivery in these scenarios.

The neonate model was developed based on a 90th-percentile neonate and consisted of 32 ellipsoidal bodies that were connected by kinematic joints. The model had a bisacromial diameter of 14.0 cm. The BP of the neonate model was modeled as a 7.5 cm nonlinear spring and tensile failure properties of the musculocutaneous (MSC) nerve of neonatal piglets, studied *in vitro* by Singh et al. (2018), were applied using the Load Displacement function (Grimm et al., 2010). The maternal pelvis model was developed as a separate multi-body system using a Computer-Aided Design based on a computed tomography scan of a 50th-percentile female gynecoid pelvis. It had an obstetric conjugate of 12.5 cm (Verspyck et al., 1999). The chosen 90th percentile size for the neonate model and 50% size for the maternal model with 12.5 cm obstetric conjugate was based on previously reported studies.

**FIGURE 1:**
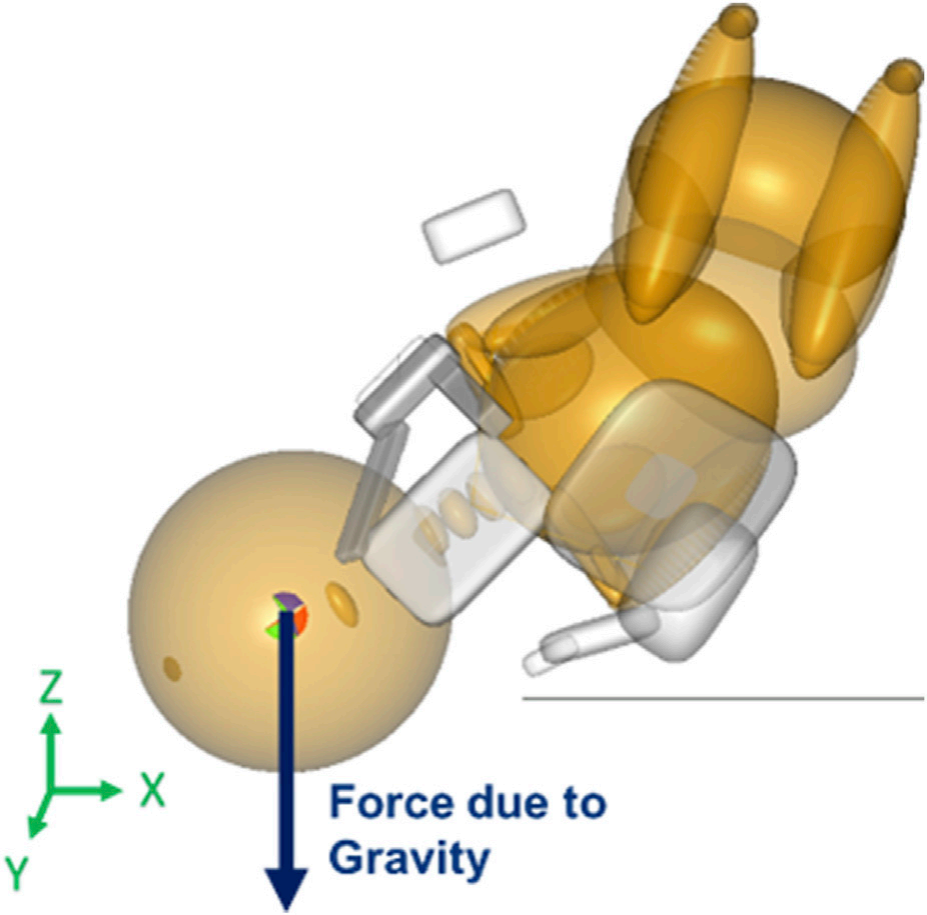
Force vector for gravity applied to the center of gravity/mass of the neonate’s head in the MADYMO model.

The neonate model was subjected to three forces. Gravity was applied along the same direction and magnitude for all simulations. It was applied to the center of gravity (coincided with the center of mass) of the head of the neonate in the Z-direction (Figure 1). The second force was maternal force which varied at two levels, namely, 82N and 129N. The maneuvers were simulated with 82N and 129N of maternal force each to represent uterine contractions and contractions with Valsalva forces, respectively (Buhimschi et al., 2001).

**FIGURE 2:**
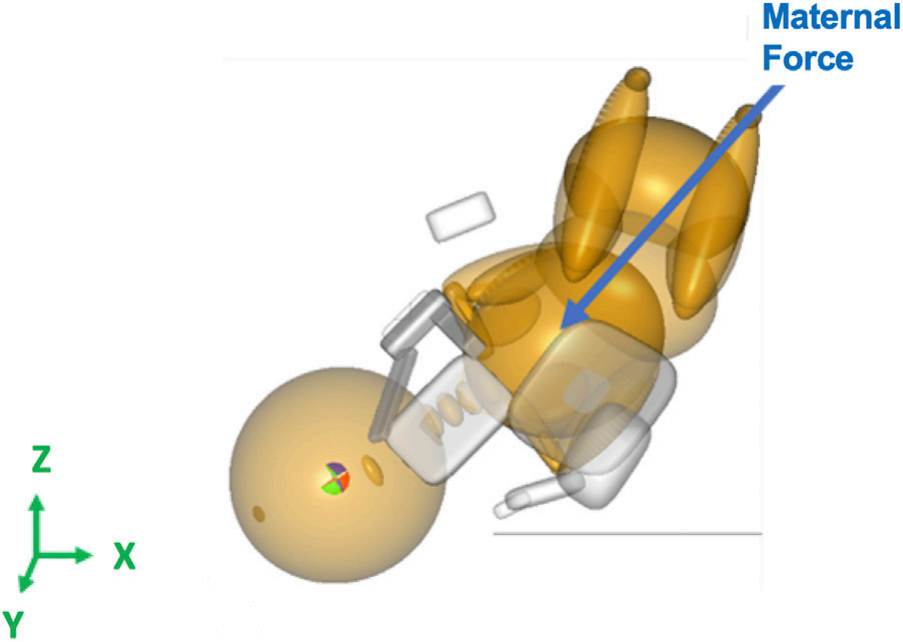
Force vector for the maternal force applied to the center of gravity of the neonate’s upper torso in the MADYMO model.

**FIGURE 3:**
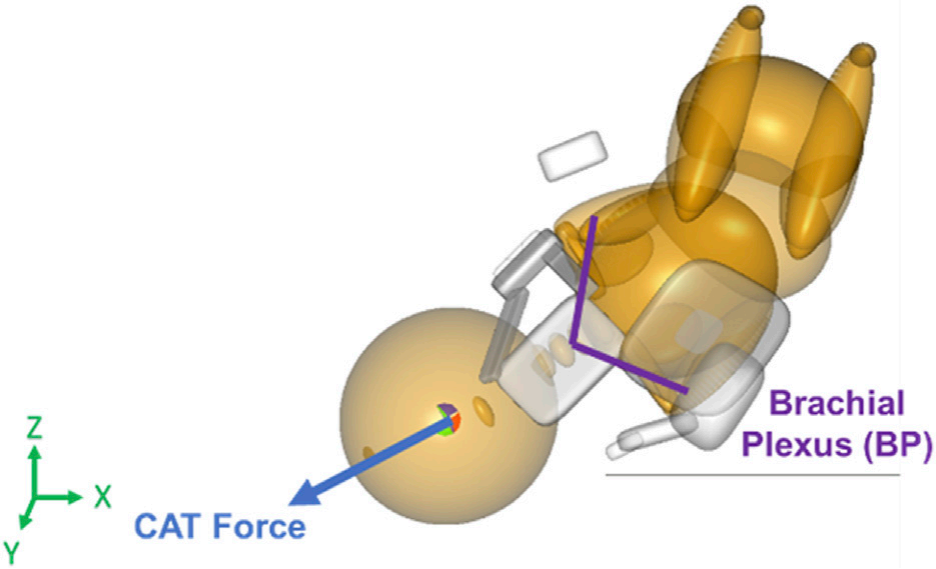
Force vector for clinician-applied traction (CAT) force applied to the center of gravity of the neonate’s head in the direction of the spine in the MADYMO model.

The maternal force was applied to the center of gravity of the neonate’s upper torso downward and outwards with respect to the birth canal at a 45° angle, along the axis of the spine of the neonate downwards in the Z-direction and out of the pelvis creating a resultant force vector as shown in Figure 2 (Grimm et al., 2010). Clinician-applied traction (CAT) delivery force was applied to the center of gravity of the neonate’s head and in the direction of the spine to prevent lateral bending of the neck of the neonate (Figure 3). In simulations where delivery was not achieved through the applied maternal force, CAT delivery force was applied starting from 0N with 5N increments (frictionless boundary condition assumed) until delivery of the anterior shoulder of the neonate was observed in the MADYMO post-processor. When delivery was achieved, the corresponding CAT force was recorded as the force required to achieve delivery. In all simulations, the resulting neonatal BP stretch during delivery was recorded.

**FIGURE 4:**
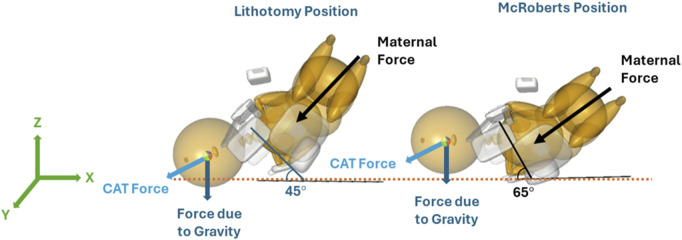
Orientation of the MADYMO models in the lithotomy (left) and McRoberts (right) positions with respect to the horizontal X-axis.

Two maternal pelvis positions, lithotomy, and McRoberts, were simulated and compared in this study. The lithotomy position was treated as the baseline position to represent the starting birthing position of the mother. The maternal pelvis and neonate were oriented at a 45° angle with respect to the horizontal x-axis. The orientation and positions of the models in the lithotomy position were adapted from a previous study (Grimm et al., 2010). In the clinic, the McRoberts position is widely used to manage shoulder dystocia by bringing the mother’s knees to her chest. The McRoberts position was implemented into the model by rotating the pelvis 20° posteriorly (about the y-axis) from the lithotomy position (Allen, 2007). A lateral view of the neonate and maternal pelvis MADYMO models in the lithotomy (left) and McRoberts (right) positions are shown in Figure 4. The lithotomy and McRoberts positions were both simulated without additional maneuvers and their results were compared.

**FIGURE 5:**
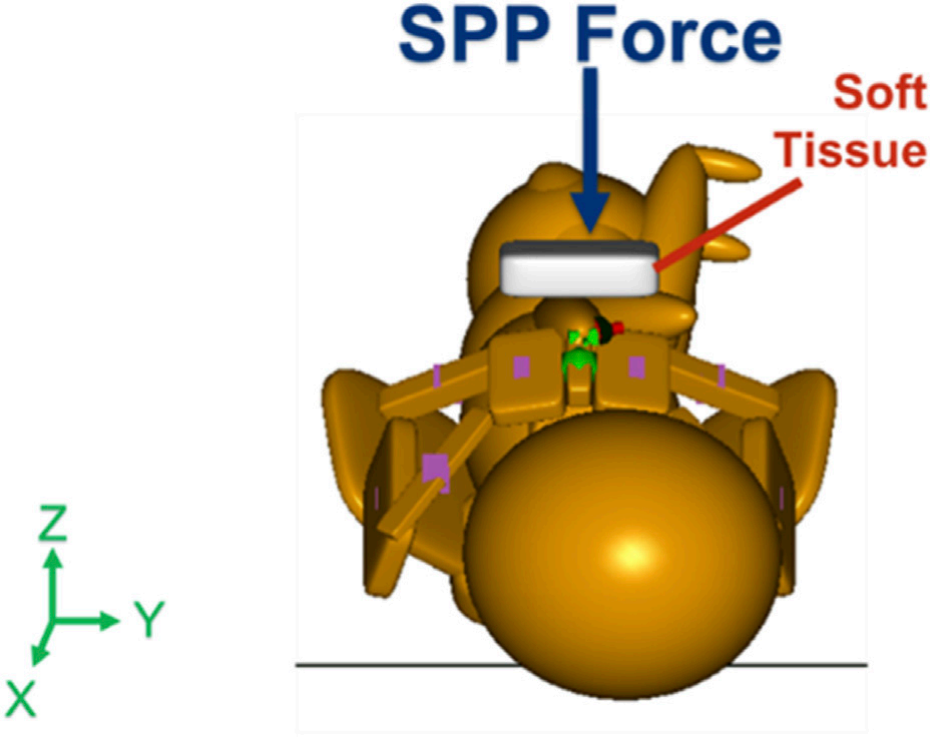
Force vector of suprapubic pressure applied by the clinician at magnitudes of 40 N, 100 N, and 140 N.

**FIGURE 6:**
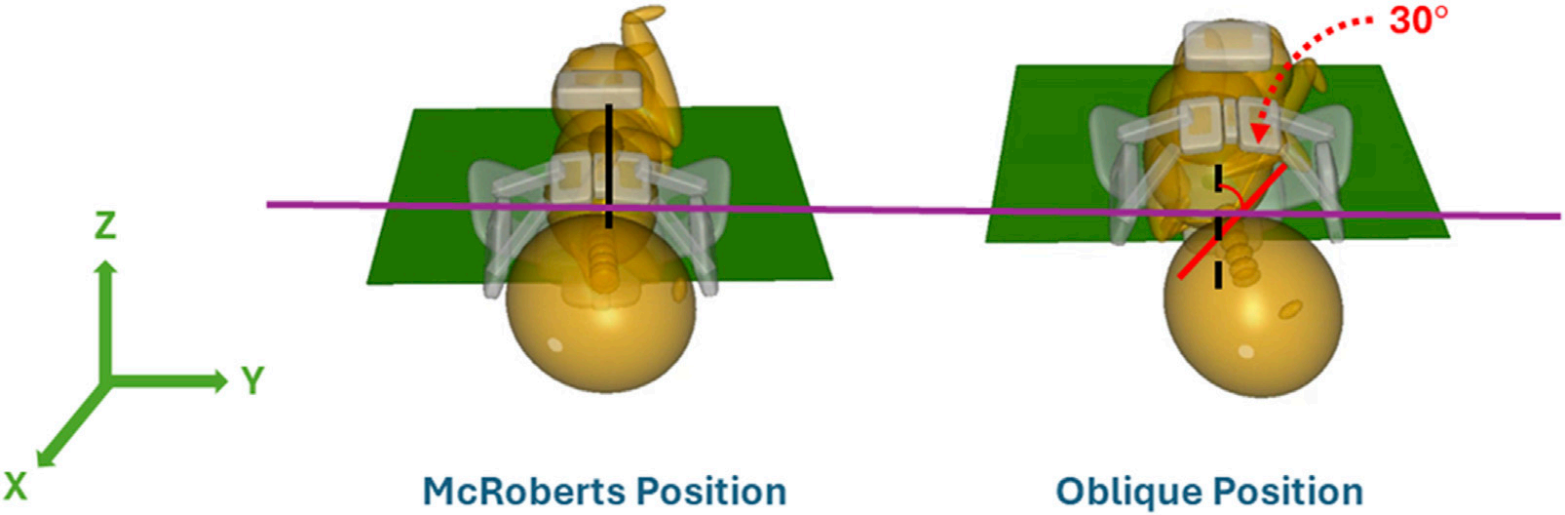
McRoberts position compared to the oblique position of the neonate model.

**FIGURE 7:**
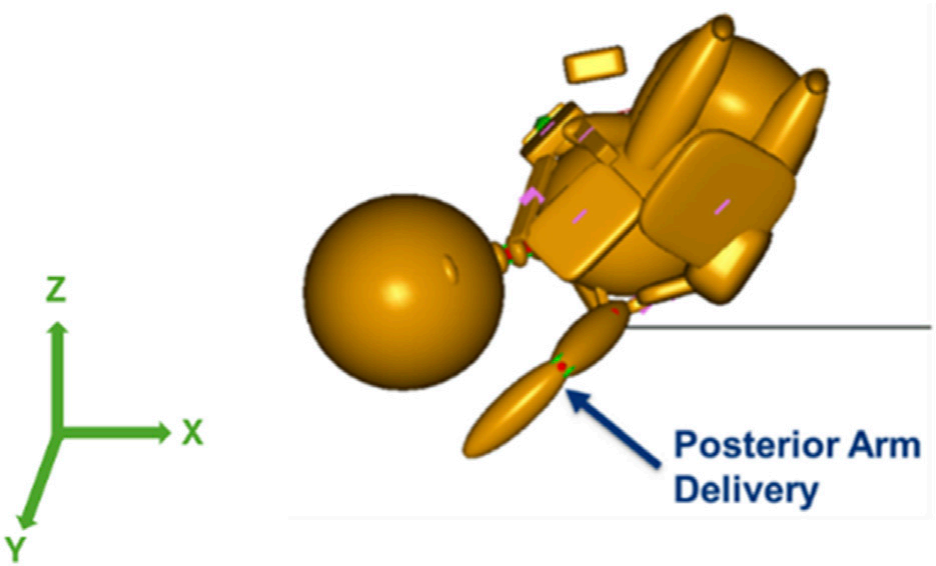
Posterior arm delivery maneuver model in which the posterior arm was delivered prior to the anterior shoulder of the neonate.

With the maternal pelvis in the McRoberts position, three neonate maneuvers were then simulated. The first neonate maneuver was the application of SPP. In the clinic, the SPP maneuver includes a clinician-applied pressure to the soft tissue superior to the symphysis pubis of the maternal pelvis with the goal of compressing or rotating the neonate’s shoulders. SPP was implemented in the MADYMO models by applying a body actuator to the soft tissue body directly superior to the maternal symphysis pubis in the Z-direction, as shown in Figure 5. The Soft tissue was represented by force-based contact characteristics in-built into the MADYMO Software. SPP was applied at three magnitudes, namely, 40 N, 100 N, and 140 N, to show the effects of varying SPP forces on BP stretch. The next maneuver that was modeled was the oblique positioning of the neonate, which represents the position of the neonate when maneuvers such as Woods’ screw or Rubin’s are completed. As shown in Figure 6, the neonate model was rotated 30° clockwise about the x-axis toward the posterior of the maternal pelvis to represent the oblique position (Gurewitsch and Allen, 2005; Allen, 2007). Finally, for simulating the posterior arm delivery maneuver, the initial position of the posterior arm of the neonate model was extended out of the maternal pelvis thereby representing the final position of the neonate when the posterior arm maneuver is completed (Figure 7).

The application of SPP, oblique positioning, and posterior arm delivery was simulated in the McRoberts position (pelvis rotated by 20°) and at two levels of maternal forces (82N and 129N). The maternal forces of 82 N and 129 N (calculations performed using the obtained clinical intrauterine pressure data and estimated ‘pelvic inlet area’) represent uterine contractions and contractions with active pushing that act as expulsive forces to deliver the neonate (Buhimschi et al., 2001). BP stretch during delivery was recorded when the neonate’s anterior shoulder cleared the symphysis pubis of the maternal pelvis in all simulated scenarios. Further, the CAT delivery force, if required for delivery was also reported.

Sensitivity Analysis: To determine the sensitivity of the models to the biomechanical properties of the BP, the BP stiffness was increased and decreased by 10% and the BP stretch during delivery and any required CAT force for delivery were recorded. The BP stiffness was changed in the lithotomy and McRoberts maternal positions at both studied maternal forces (82N and 129N) without the neonate-focused maneuvers.

## 3 Results

Delivery of the neonate was simulated in the lithotomy and McRoberts positions to show the effects of the McRoberts maneuver on the neonatal brachial plexus and to determine any required CAT delivery force. Required CAT delivery forces and the resulting BP stretch observed in the lithotomy and McRoberts positions with two levels of maternal force are shown in Table 1.

**TABLE 1:** Required clinician-applied traction force to achieve delivery and resulting brachial plexus stretch for the lithotomy and McRoberts positions at 82 N and 129 N applied Maternal force.

Maternal force (N)	Lithotomy		McRoberts	
	CAT force (N)	BP stretch (%)	CAT force (N)	BP stretch (%)
82	50	21.21	25	19.47
129	0	21.89	0	21.83

The results show a reduction in both CAT delivery force and BP stretch when the mother is moved from lithotomy into the McRoberts position at 82 N maternal force. This indicates that less force is required by the clinician to deliver the neonate and the BP of the neonate experiences less stretch due to the change in position of the pelvis. At 129 N maternal force, no CAT delivery force was required, and the BP stretch-induced during delivery was similar for the lithotomy and the McRoberts positions.

With the models in the McRoberts position, neonate maneuvers were simulated to observe the effects of these maneuvers on neonatal brachial plexus during delivery, Furthermore, the required CAT delivery force for delivery was also investigated when the applied maternal forces could not deliver the neonate. CAT delivery forces and BP stretch that resulted in delivery during various neonate maneuvers with two pre-determined levels of maternal force are shown in Table 2.

**TABLE 2:** Required clinician-applied traction (CAT) force to achieve delivery and resulting brachial plexus stretch for each maneuver simulation applied in the McRoberts position.

Neonate maneuver	Maternal force: 82 N		Maternal force: 129 N	
	CAT force (N)	BP stretch (%)	CAT force (N)	BP stretch (%)
McRoberts only	25	19.47	0	21.83
SPP 40 N	15	17.96	0	19.85
SPP 100 N	0	15.84	0	16.72
SPP 140 N	0	14.06	0	15.01
Oblique	0	8.86	0	10.82
Posterior Arm	0	7.73	0	8.06

At 82N maternal force, the neonate-focused maneuvers (SPP, Oblique, and Posterior Arm) reported a further decrease in the CAT force and the resulting BP stretch when compared to McRoberts only condition. In the case of the SPP maneuver, increasing SPP from 40N to 100N reported no required CAT force for delivery at 100N along with decreased BP stretch from 17.96% to 15.84%, respectively. Also, increasing the SPP from 100N to 140N, further decreased the BP stretch while no additional CAT delivery force was required. The Oblique positioning further decreased the BP stretch to 8.86%, and the posterior arm delivery of the neonate resulted in the least amount of BP stretch at 7.73%. No CAT forces were required during these maneuvers (Oblique and Posterior Arm). The simulations at 129N maternal force reported similar trends of decrease in the amount of BP stretch during delivery except no CAT forces were required during all simulations including McRoberts only, SPP, Oblique, and Posterior Arm (Table 2).

In the lithotomy and McRoberts positions, the stiffness of the modeled BP was changed by +/−10% to determine whether the models were sensitive to BP properties, and these results are displayed in Table 3.

**TABLE 3:** Sensitivity analysis to determine the effects of BP stiffness on BP stretch in the lithotomy and McRoberts positions.

Maternal force (N)	Lithotomy		McRoberts	
	CAT force (N)	BP stretch (%)	CAT force (N)	BP stretch (%)
+10% BP Stiffness				
82	50	20.595	25	19.04
129	0	21.258	0	20.996
−10% BP Stiffness				
82	50	23.291	25	19.878
129	0	23.167	0	22.395

## 4 Discussion

Shoulder dystocia is an obstetric emergency that prevents the delivery of the anterior shoulder of the neonate due to the obstruction created by the symphysis pubis of the maternal pelvis (Ouzounian, 2016; Dash et al., 2018; Menticoglou, 2018). Attempts to alleviate the obstruction can lead to overstretching of the BP that could result in NBPP (Dunbar et al., 2021). Effects of maternal/clinician applied forces and clinical maneuvers on neonatal BP are ethically and technically challenging to study in clinical settings. Computational models like MADYMO serve as a promising alternative (Gonik et al., 2003b; Zhang et al., 2003; Grimm et al., 2010). In this study, MADYMO was utilized to study the effects of maternal/clinician-applied forces and various maneuvers, that are widely employed for the management of shoulder dystocia, on the BP stretch (Zimerman et al., 2018). The maneuvers selected for this study are practiced in a particular sequence by clinicians to prepare for shoulder dystocia (Dash et al., 2018). In each of the simulations, two levels of maternal force, which have been calculations performed using the clinically measured maternal intrauterine pressure data and estimated ‘pelvic inlet area’, were applied where 82 N simulated the uterine contractions and 129 N simulated contractions with Valsalva forces (Buhimschi et al., 2001). If required, the CAT delivery force was applied to the head of the neonate and increased incrementally (5N increments) in the simulations until delivery of the anterior shoulder was observed. For all studied simulations, the BP stretch during delivery was then recorded.

The results in Table 1 show the comparison between the maternal positions of lithotomy and McRoberts at the two levels of applied maternal forces. As the applied maternal force increased from 82 N to 129 N, the required CAT force decreased. This relationship between the maternal force and CAT delivery force in both positions is expected as the greater expulsive force applied to the neonate’s torso requires less CAT delivery force applied to the head during delivery. There was also an increase in BP stretch due to the increase in the applied maternal force. This trend was observed in both the lithotomy and McRoberts positions and with each neonate maneuver. This relationship was due to the increasing expulsive force acting on the neonate trunk thereby increasing the contact force between the anterior shoulder of the neonate and the maternal symphysis pubis and therefore resulting in increased BP stretch (Grimm, 2021).

The McRoberts maneuver was implemented in MADYMO by rotating the pelvis and neonate models 20° posteriorly from the lithotomy position. The results showed a decrease in the required CAT delivery force and BP stretch in the McRoberts position compared to the lithotomy position, at 82N maternal force (Allen, 2007). The McRoberts maneuver flattens the sacral promontory to allow more tolerance for the neonatal shoulders within the pelvic inlet (Gurewitsch and Allen, 2005; Allen, 2007; Grimm et al., 2010). This decreases both the CAT delivery force required to deliver the neonate and the stretch on the neonate’s BP. These results of the McRoberts maneuver are supported by the success rate of alleviating approximately 42% of shoulder dystocia cases, reported in the literature (Gherman et al., 1997). Previous studies investigating the effects of the McRoberts maneuver with computational modeling also observed a decrease in required delivery force, in our case CAT since the maternal force was the same at 82N, and BP stretch compared to the baseline lithotomy position (Mazzanti, 1959). A previously reported physical model study, using a tactile sensing glove and neck extension sensors, also reported a decrease in BP stretch due to the McRoberts maneuver (Gonik et al., 1989).

With the maternal pelvis model maintained in the McRoberts position, the application of SPP forces was simulated (McFarland et al., 1996). SPP forces were implemented in the MADYMO model by applying a force to the soft tissue structure located superior with respect to the maternal symphysis pubis, directly over the shoulder of the neonate (Lok et al., 2016). The SPP force vector was applied along the -Z-axis, at 40 N, 100 N, and 140 N. The results of the application of SPP in the MADYMO model are shown in Table 3. As the applied SPP force increased, the required CAT delivery force and resulting BP stretch decreased. The downward-directed SPP force contributed to the delivery of the shoulders of the neonate, therefore decreasing the CAT delivery force required for the delivery such that no CAT delivery force was required at SPP of 100 N and 140 N at both 82 N and 129 N maternal forces (Penney and Perlis, 1992). SPP force also helped reduce BP stretch by lowering the anterior shoulder under the pubic bone and as the applied SPP forces increased from 40 N to 140 N, the resulting BP stretch during delivery decreased.

The oblique position was modeled in MADYMO by rotating the mid-coronal plane of the neonate model by 30° toward the mother’s posterior. This represented rotational maneuvers, and the neonate model was positioned such that the rotation had just been completed by the clinician at the start of the simulation (Hoffman et al., 2011). The bisacromial diameter of the neonate was in alignment with the obstetric conjugate of the maternal pelvis, which is the smallest measurement of the pelvis inlet until the neonate was rotated via rotational maneuvers. The oblique position aligned the shoulders of the neonate with the oblique diameter. The oblique diameter allows for more space for the shoulders of the neonate to deliver, alleviating the obstruction with the symphysis pubis. The simulation resulted in a lower CAT delivery force and resulting BP stretch, at both 82 N and 129 N maternal forces when compared to McRoberts alone and SPP simulations. Literature has reported the effectiveness of rotational maneuvers, represented by the oblique positioning in these models, due to the alignment of the neonate’s bisacromial diameter with the oblique diameter of the pelvis (i.e., the largest dimension of the pelvic inlet) (Gurewitsch and Allen, 2005; Allen, 2007).

The last neonate maneuver that was simulated was the delivery of the posterior arm. It was modeled in MADYMO such that the maneuver had just been performed by the clinician and the simulation started with the posterior arm already delivered. By delivering the posterior arm prior to the shoulders of the neonate, the bisacromial diameter was reduced by an approximate arm width (Menticoglou, 2018; Gachon et al., 2016). With posterior arm delivery, no CAT delivery force was required at both 82 N and 129 N maternal forces, and the resulting BP stretch during delivery was lowest among all simulated neonate-focused maneuvers. Published literature also reports the oblique and posterior arm delivery maneuvers to be the most effective in alleviating the obstruction due to shoulder dystocia and reducing BP stretch, although they are more invasive and challenging maneuvers (McFarland et al., 1996).

The trends that resulted from the simulations modeled in this study provide insight into the effectiveness of clinical maneuvers and subsequent BP stretch. While computational modeling can be advantageous for modeling clinical scenarios that cannot be studied and measured in actual clinical settings, some limitations of the models exist (Cechova et al., 2021). In this study, frictionless contact conditions were assumed. The sensitivity of the model was also only validated for the used BP properties. Furthermore, patient specificity including size, geometry, and material properties varies among cases that were not accounted for in this model. Therefore, magnitudes of force and BP stretch might not be accurate but trends in the results can be translated to offer an understanding of the effects of studied maneuvers. Another limitation of this study is that each maneuver was simulated independently whereas they are performed sequentially in the clinical settings (McFarland et al., 1996). Thus, the model does not show the effects of multiple maneuvers being performed in a clinical setting.

Future studies with this model should apply *in vivo* biomechanical properties to the BP of the neonate model to enhance the biofidelity of the neonate model (Balasubramanian et al., 2016; Singh et al., 2019a; Singh et al., 2021; Singh et al., 2020a; Singh et al., 2022; Majmudar et al., 2021; Orozco et al., 2021). Furthermore, computational studies of shoulder dystocia and BP stretch could utilize Finite Element Models (FEM) to include soft tissue structures of the mother and anatomical accuracy of the neonate’s shoulder and neck (Singh and Ferry, 2019; Singh et al., 2019b). These models can then be adjusted to create patient-specific FEM and help provide training and planning for complicated deliveries. The models can serve as useful training tools for clinicians to better understand the mechanisms of injury during complicated delivery and improve patient outcomes (Singh and Ferry, 2015; Singh and Ferry, 2019; Singh et al., 2018; Singh et al., 2020b).

## Data Availability

The original contributions presented in the study are included in the article/supplementary material, further inquiries can be directed to the corresponding author.
